# Elevated ALOX12 in renal tissue predicts progression in diabetic kidney disease

**DOI:** 10.1080/0886022X.2024.2313182

**Published:** 2024-02-12

**Authors:** Meixi Wang, Jingjing Wang, Jinni Wang, Yonggui Wu, Xiangming Qi

**Affiliations:** a Department of Nephropathy, The First Affiliated Hospital of Anhui Medical University, Hefei, China; b Center for Scientific Research of Anhui Medical University, Hefei, China

**Keywords:** Diabetic kidney disease, ALOX12, bioinformatics, transcriptome

## Abstract

Diabetic kidney disease (DKD) is one of the major causes of end-stage renal disease and one of the significant complications of diabetes. This study aims to identify the main differentially expressed genes in DKD from transcriptome sequencing results and analyze their diagnostic value. The present study sequenced db/m mouse and db/db mouse to determine the ALOX12 genetic changes related to DKD. After preliminary validation, ALOX12 levels were significantly elevated in the blood of DKD patients, but not during disease progression. Moreover, urine ALOX12 was increased only in macroalbuminuria patients. Therefore, to visualize the diagnostic efficacy of ALOX12 on the onset and progression of renal injury in DKD, we collected kidney tissue from patients for immunohistochemical staining. ALOX12 was increased in the kidneys of patients with DKD and was more elevated in macroalbuminuria patients. Clinical chemical and pathological data analysis indicated a correlation between ALOX12 protein expression and renal tubule injury. Further immunofluorescence double staining showed that ALOX12 was expressed in both proximal tubules and distal tubules. Finally, the diagnostic value of the identified gene in the progression of DKD was assessed using receiver operating characteristic (ROC) curve analysis. The area under the curve (AUC) value for ALOX12 in the diagnosis of DKD entering the macroalbuminuria stage was 0.736, suggesting that ALOX12 has good diagnostic efficacy. During the development of DKD, the expression levels of ALOX12 in renal tubules were significantly increased and can be used as one of the predictors of the progression to macroalbuminuria in patients with DKD.

## Introduction

Global mortality rates of chronic kidney disease (CKD) increased by 41.5% between 1990 and 2017. According to predictions, the prevalence of CKD will rise quickly and pose a global health care issue [1,2]. Diabetic kidney disease (DKD) is a complication that affects 40% of diabetes mellitus type 2 (DM2) and 30% with diabetes mellitus type 1 (DM1), and it is the main cause of CKD. The prevalence of patients with DKD is anticipated to rise in tandem with global diabetes [3]. In 2015, an estimated 415 million people worldwide had diabetes; by 2040, the prevalence will increase to 642 million [3]. Therefore, it is urgent to find early diagnostic biomarkers for DKD to provide early treatment.

Numerous cells and cytokines participate in the complicated pathophysiology of DKD [4]. The outcome of all cells is survival, death, and transdifferentiation. All three aspects are regulated by cellular programmed mechanisms and accompanied by genetic alterations. Genomics focuses on the structure, function, evolution, localization, and editing of genomes and their effects on the organism [5]. Genomic assays have been previously used. However, technology has advanced, and thus, genomic assays can be an important tool for mechanistic studies [6]. Therefore, in the present study, the arachidonic acid (AA) cyclooxygenase pathway was identified as an important metabolic pathway associated with cell survival or death in DKD by genomic analysis.

The AA cyclooxygenase pathway plays a crucial part in the pathogenesis of diabetes and its related kidney consequences [7,8]. There are three metabolic pathways of AA: the cyclooxygenase pathway, which results in prostaglandins and thromboxanes; the lipoxygenase (LO) pathway, which results in LOs such as 5-, 12-, and 15-hydroxyeicosatetraenoic acids (HETEs) and leukotrienes; and the cytochrome P-450 cyclooxygenase pathway, which results in epoxides and other products [9].

These metabolites are potent autocrine and paracrine biologically active mediators that are widely involved in a number of physiological and pathological processes. The oxidative metabolism of polyunsaturated fatty acids is carried out by LOs, which are categorized as 5-, 8-, 12-, and 15-LOX (LO) [10]. 12-LOX activation produces 12-(S)-hydroxyeicosatetraenoic acid (12(S)-HETE), which is a product of AA [11]. In the current study, 12-LOX was found to be significantly increased in rat mesangial cells (MCs) exposed to high glucose (HG) [12]. However, in humans, the corresponding studies are fewer and more controversial. There was no significant correlation between the ALOX12 (arachidonic acid 12-LO) polymorphism and proteinuria in patients with diabetes at various stages of CKD [13]. Even so, in two investigations, ALOX12 rs1126667 was linked to increased proteinuria in patients with DM2 [14], and proteinuria was immediately brought on by 12(S)-HETE [15]. Additionally, ALOX12 was more highly associated with vascular effects, such as hypertension and atherosclerosis, and more likely to occur in DM2 than in DM1 patients [16]. Therefore, further research on the clinical significance of ALOX12 in type 2 DKD is needed. In this study, the clinical value of ALOX12 in the progression of proteinuria in DKD was investigated in depth in the context of clinical biochemical and pathological data in type 2 DKD patients from a genomic perspective based on transcriptome sequencing results and bioinformatics analysis.

## Materials and methods

### Transcriptome sequencing

The control group included 6–8-week-old db/m male mouse (*n* = 6), while the diabetes animal model group included 6–8-week-old db/db male mouse (*n* = 6). All animals were purchased from the Institute of Simulated Animal Research, Nanjing University, Nanjing, China, and both groups received proper nutrition for 20 weeks. Kidney specimens from three mice were randomly selected from each group after collecting kidney tissue and then sent to Shanghai OEE Biological Company (Shanghai, China) for perfecting the transcriptome sequence.

### Identification of differentially expressed genes (DEGs)

Differences in the two groups were investigated using the Wilcoxon test. The limma software package was used for differential expression analysis in R software (R Foundation for Statistical Computing, Vienna, Austria), using |log2fold change (FC)| > 1 and *p* < .05 to identify DEGs in kidney tissues from the model and control groups. The heatmap and Enhanced Volcano software packages were used to visualize the DEGs.

### Functional enrichment analysis

Gene Ontology (GO) and Kyoto Encyclopedia of Genes and Genomes (KEGG) enrichment studies were carried out by the 'clusterProfiler' package, and an adjusted *p* value < .05 was taken to reflect statistical significance; ggplot2 was used to create the plots. The protein–protein interaction (PPI) network analysis was constructed using the STRING (http://string-db.org) database, and the top 10 hub genes were filtered using the MCODE plugin of Cytoscape.

### External dataset validation

We chose the GPL8300 and GSE1009 datasets, which include three healthy patient samples and three DKD patient samples, and the GPL1355 and GSE7253 datasets, which include six diabetic rat kidney samples and six nondiabetic rat kidney samples, as validation sets (http://www.ncbi.nlm.nih.gov/geo/).

### Real-time polymerase chain reaction (RT-PCR) of mouse kidney tissue

RNA from mouse kidney tissue was extracted. RT-PCR was performed on total RNA and was then reverse transcribed to cDNA using Hifair II SuperMix plus. cDNA was amplified using a T100 thermal cycler (Bio-Rad, Hercules, CA). Amplification specifications were established as follows: 95 °C predenaturation for 5 min, 95 °C denaturation for 10 s, and annealing at 60 °C for 30 s for 40 cycles. cDNA was subjected to qPCR using ABI QuantStudio 5 (Applied Biosystems, Foster City, CA). The β-actin gene was used as an endogenous control for mRNA. Primer sequences are shown in Supplementary Table 2. The △Ct technique was used to determine the relative expression of the ALOX12 gene. RNA variations among db/m and db/db mouse were calculated using the 2^−△△CT^ method.

### Volunteers in the study

Serum and clean midstream urine were collected from six healthy patients from the physical examination center, six patients with minimal change disease (MCD), and 12 patients with DKD. Additionally, 10 participants with MCD confirmed by renal biopsy and 42 patients with biopsy-confirmed DKD were enrolled in the study. For comparison, para-cancerous kidney tissue was taken from six participants with renal cancer in our institution as controls. The First Affiliated Hospital of Anhui Medical University's Medical Ethics Committee approved the trial (PJ2022-13-10), conducted from January 2019 until September 2022. All volunteers signed an informed permission before collecting clinical information and tissue samples.

The inclusion criteria for the DKD group were as follows: (1) age 18–80 years; (2) diabetes mellitus, with diagnostic indicators including random blood glucose level of ≥11.1 or fasting plasma glucose (FPG) level ≥7 mmol/L; (3) DKD diagnosed by renal biopsy. The exclusion criteria included the following: (1) other forms of diabetes, including type 1 diabetes, owing to autoimmune disease, chronic pancreatitis, surgery, or drugs; (2) acute myocardial infarction, congestive heart failure, or cerebrovascular illness; liver failure with alanine aminotransferase levels more than double the outer bound of normal; urolithiasis, cancer, urinary tract disease due to infection; and acute complications of diabetes such as ketoacidosis.

The exclusion criteria for normal control (NC) group and MCD group were as follows: (1) diabetes mellitus [17]; (2) severe hypertension; (3) hepatic impairment with secondary renal disease, heart disease, malignant hypertension, myocardial infarction, cerebrovascular accident, infection, and liver failure with alanine aminotransferase levels more than double the outer bound of normal; (4) pregnancy or lactation; (5) in NC group, patients who had received chemotherapy or had abnormal changes in kidney-related markers (serum creatinine or glomerular filtration rate) were also included [18].

### Determination of ALOX12 protein expression in human serum and urine

Five milliliters of venous blood and 5 mL clean mid-stream urine were obtained from each patient. Serum and urine ALOX12 concentrations were measured using enzyme-linked immunosorbent assay (ELISA) kits, in accordance with the manufacturer's instructions. Using an enzyme marker reader with many functions, fluorescent signals were recorded [18].

### Clinical biochemistry and pathology data collection

The baseline characteristics of age, sex, and U-ACR (urine-albumin/urine creatinine ratio) were obtained. Height and weight measurements were obtained upon check-in. The DKD group was further divided into two groups: U-ACR <300 mg/g (microalbuminuria and normoalbuminuria group (DKD1 group)) and U-ACR ≥300 mg/g (macroalbuminuria group (DKD2 group)) [19]. On the morning of the second day after admission, biochemical tests were performed after 6–8 h of fasting, and clinical biochemical data were collected from the patients. We also measured essential renal pathological indicators, such as glomerular volume, MC proliferation, stromal expansion, tubular epithelial cell atrophy, and tubular dilatation, from renal puncture specimens from patients for further analysis. Each biopsy contained a minimum of eight glomeruli for evaluation. Pathological features of DKD were assessed by a pathologist. Glomerulopathy was evaluated by Tervaert's classification of renal histopathology in the DKD group. Class I: thickening of glomerular basement membrane. Class II: tethered membrane dilatation, mild; (IIa) or severe (IIb); class III: tuberous sclerosis (Kimmelstiel-Wilson lesion); class IV: advanced diabetic glomerulosclerosis. Scoring criteria for interstitial fibrosis and tubular atrophy (IFTA) scale: 0 for none; 1 for <25%; 2 for 25–50%; 3 for >50%. Scoring criteria for interstitial inflammation: 0 for absence; 1 for inflammation only infiltrating the fibrotic area; 2 for inflammation infiltrating the non-fibrotic area accordingly. Scoring criteria for arteriolar hyalinosis: 0 for absence; 1 for occasional occurrence; 2 for frequent occurrence. Scoring criteria for atherosclerosis: 0 for no intimal thickening, 1 for intimal thickening not exceeding the middle membrane, and 2 for intimal thickening exceeding the middle membrane [18].

### Immunohistochemistry (IHC) and immunofluorescence (IF)

Histopathological changes in the mouse kidney were observed using HE and PAS staining. According to the manufacturer's directions, the staining was done.

Kidney puncture specimens from patients and fresh kidney tissue from mouse were immediately cut into paraffin blocks. Then, they were successively sliced into 3 μm-thick sections and attached to microscope slides. After dewaxing, the plates were placed in boiling sodium citrate, heated for 5 min for repair, and cooled to room temperature. Blocking was performed using an endogenous peroxidase blocker for 30 min at 37 °C, followed by reduction using 10% goat serum for 30 min at the same temperature. Subsequently, the plates were incubated overnight at 4 °C with ALOX12 (1:200 dilution, Abclonal, Wuhan, China, cat. number: A14703) and NGAL (1:200 dilution, Abcam Biotechnology, Cambridge, UK, cat number: ab23477 for human tissues; 1:100 dilution, Affinity Biosciences, Kwun Tong, Hong Kong, China, cat number: DF6816 for animal tissues) primary antibodies. After washing, tissue slices were incubated for 30 min at 37 °C with secondary antibodies. Finally, to complete the chromogenic reaction, diaminobenzidine (DAB) was added, and cell nuclei were stained with hematoxylin. Brown staining indicates a positive result. The quantity of immunostaining was assessed with ImageJ analysis software (Bethesda, MD). For IF staining, anti-ALOX12 was incubated overnight at −4 °C with renal tubule-resident cell markers (anti-NGAL, anti-AQP1, anti-CD28K, and anti-AQP3), followed by incubation with fluorescently labeled secondary antibodies for 1 h at 37 °C. Nuclei were stained with DAPI. Sections were observed under an inverted fluorescence microscope (Zeiss Spot, Carl Zeiss Ltd., North York, Canada) and photographed [20].

### Data analysis

R 4.0.3 (https://www.R-project.org/) was used to examine and visualize the transcriptome sequencing results, and SPSS 22.0 (IBM Corporation, Armonk, NY) was used for data analysis. PASS 15.0 was used to determine the sample size, and ImageJ (Bethesda, MD) was used to examine the image data. Additionally, mapping was performed using GraphPad Prism 8 software (GraphPad Software Inc., San Diego, CA). Normally distributed data are presented as the mean ± SD and were subjected to parametric tests (two groups, Student's *t*-test; >2 groups, one-way analysis of variance (ANOVA)). Nonnormally distributed data are expressed as the median (interquartile range). Nonparametric tests (<2 groups, Mann–Whitney's test; >2 groups, Kruskal–Wallis ANOVA to examine differences between groups, followed by the Dunn–Bonferroni test) were used to investigate the nonnormally distributed data. The Chi-square test was used to assess categorical variables. Pearson's or Spearman's test was used to determine the linear connection of numerous variables with ALOX12 histochemical results. In addition, sensitivity, specificity, and area under the curve (AUC) were calculated using receiver operating characteristic (ROC) curves to measure diagnostic accuracy, and the significance of DKD-related factors in clinical diagnosis was analyzed and assessed. *p* < .05 indicates statistical significance.

## Results

### Identification of DEGs in transcriptome sequencing data

**Figure 1.**
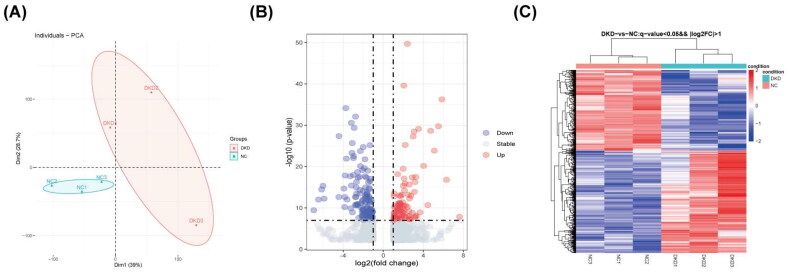
Identification of DEGs in transcriptome sequencing data. (A) PCA of sequencing results. (B) Volcano plot of the DEGs. (C) Heat map of the DEGs.

Two groups of specimens were grouped separately with high heterogeneity and good dispersion according to principal component analysis (PCA) (Figure 1(A)). A total of 1604 DEGs were identified from the sequencing data of the transcriptome. Of these, 943 in the DKD group were upregulated genes, and 661 were downregulated genes. These DEGs are shown on the volcano map (Figure 1(B,C)).

### Functional enrichment analysis and pathway analyses

**Figure 2.**
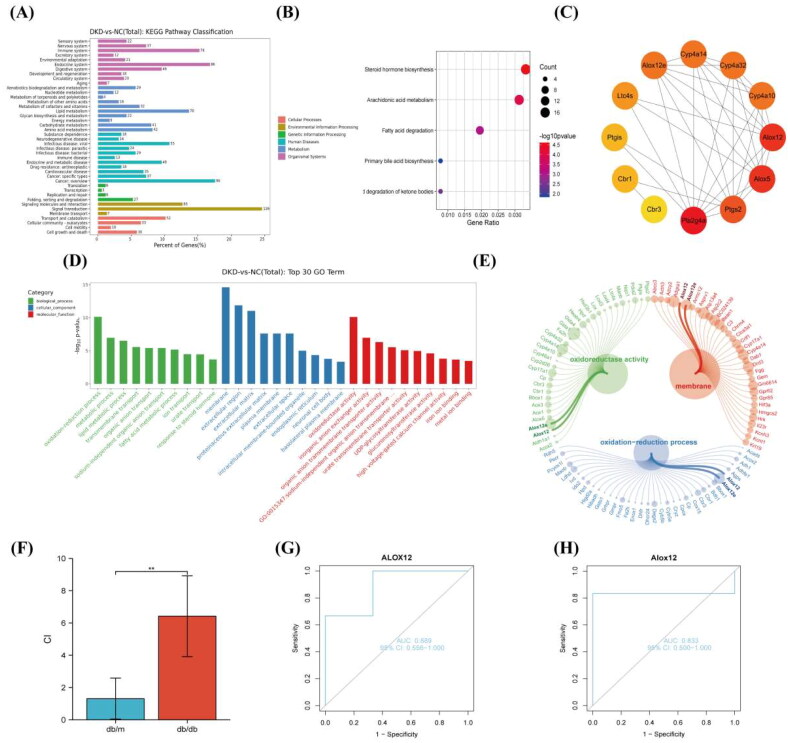
Functional enrichment analysis and pathway of DEGs. (A) KEGG enrichment analysis. (B) KEGG enrichment analysis in the lipid metabolism pathway. (C) PPI network analysis in the AA metabolism pathway. (D) GO enrichment analysis. (E) Bubble plot. (F) RT-PCR detection of ALOX12 mRNA in db/m and db/db mouse models, **p < .01. (G, H) The diagnostic efficacy of ALOX12 for DKD was verified using ROC curves in dataset GSE1009 and dataset GSE7253.

GO and KEGG analyses were performed for the DEGs to determine their relevant pathways and functions (Figure 2(A,D)). According to the KEGG pathway classification, the most significantly enriched metabolic pathways were mostly those of lipid metabolism. Renal failure and DKD may be caused by renal lipotoxicity due to an imbalance in lipid metabolism [21]. Therefore, we chose every lipid metabolism pathway for KEGG analysis to further investigate the pathological processes of DKD (Figure 2(B)). The KEGG analysis indicated that the AA metabolism pathway was the most highly significant lipid metabolism pathway and plays a role in diabetes and its associated kidney damage [7,8,22]. As a result, we decided to focus our subsequent research on the AA metabolic pathway.

We conducted PPI analysis of up-regulated DEGs in the AA metabolism pathway (Figure 2(C)). We discovered that the hub genes included the ALOX12 gene and ALOX12e gene, and these were considered the main contenders. Moreover, ALOX12 and ALOX12e were present in the first pathway of BP analysis, CC analysis and MF analysis in GO analysis (Figure 2(E)). ALOX12e is an isoform of ALOX12 [23], so we selected ALOX12 for our follow-up study. Since proteinuria and ALOX12 polymorphisms were not correlated in a prior study [13], we examined ALOX12 from a pathological angle to better understand its clinical importance. To confirm whether the genes were differentially expressed, we again validated the expression of ALOX12 mRNA in db mouse kidney tissue using RT-PCR (Figure 2(F)).

### External dataset validation

To initially verify the significance of ALOX12 in DKD, we analyze ALOX12 gene expression in the GSE1009 and GSE7253 datasets and to plot ROC curves. The AUC value for ALOX12 was 0.889 (95% CI 0.556–1.000) in GSE1009 (Figure 2(G)) and 0.833 (95% CI 0.400–1.000) in GSE7253 (Figure 2(H)). Therefore, based on the available analyses, ALOX12 has high diagnostic value in DKD samples. We tentatively hypothesize that ALOX12 may be a biomarker for diagnosing DKD.

### Histopathological level and ALOX12 level in mouse kidney tissues

**Figure 3.**
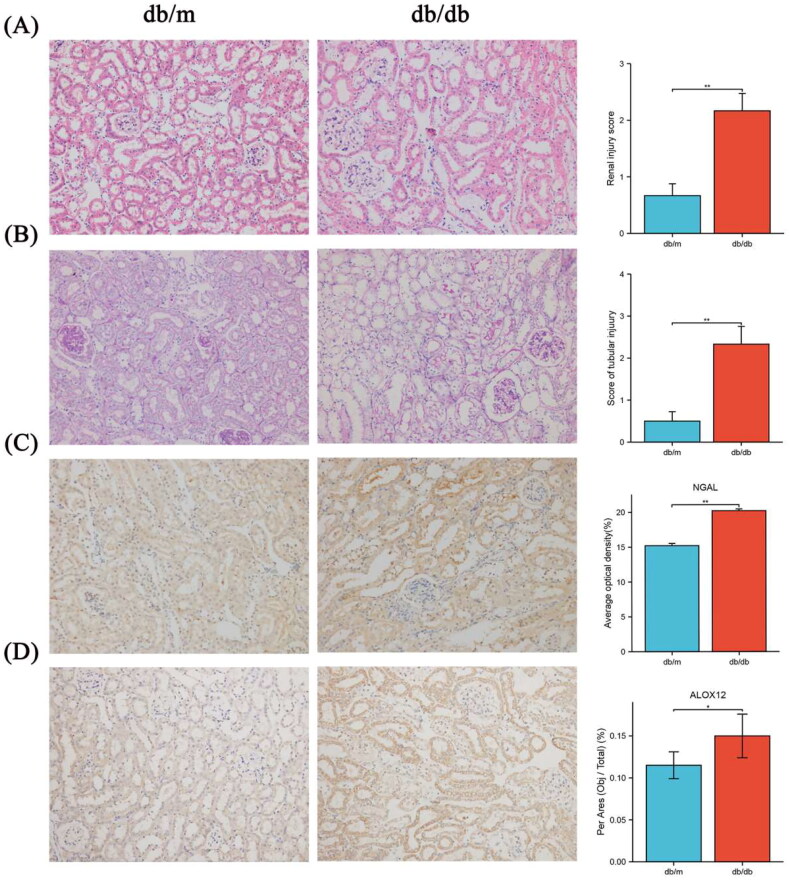
Pathological staining and ALOX12 expression in mouse kidney tissues. (A) H&E staining in db/m and db/db kidney tissue. (B) PAS staining in db/m and db/db kidney tissue. (C) IHC detection of NGAL protein expression in db/m and db/db kidney tissue. (D) IHC detection of ALOX12 protein expression in db/m and db/db kidney tissues. Scale bar = 50 μm. *p < .05, **p < .01.

Based on pathological staining, the tubular injury score, amount of glycogen deposition, and NGAL levels were increased in db/db mouse (Figure 3(A–C)), indicating that tubular damage occurred in db/db mouse. The results confirmed that the amount of ALOX12 was considerably higher in db/db mouse. ALOX12 expression was subsequently validated at the protein level using histochemical findings in mouse kidneys (Figure 3(D)). Renal function data for each group of mice are shown in Supplementary Table II.

### ALOX12 protein expression in human serum, urine and kidney tissues

**Figure 4.**
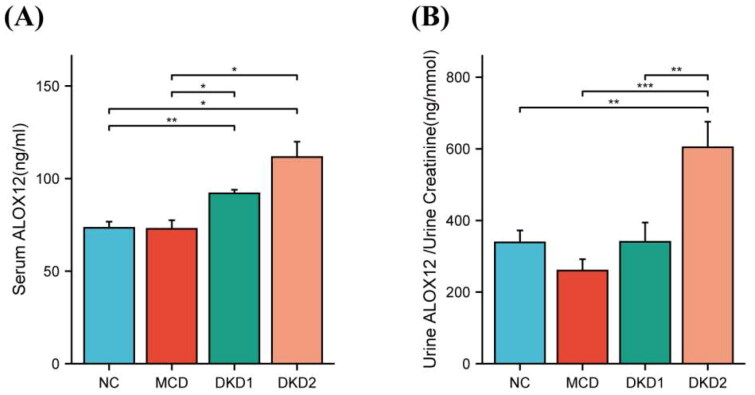
Expressions of ALOX12 in the body. (A) ALOX12 levels in serum. (B) ALOX12 levels in urine. *p < .05, **p < .01, and ***p < .001.

First, to determine the metabolism of ALOX12 in the body, we measured the levels of ALOX12 in the serum and urine in each group. We found a noticeable rise in ALOX12 levels in the serum of DKD patients compared to control individuals, but there was no difference in these levels between the DKD1 and DKD2 groups (Figure 4(A)). The DKD2 group had greater urine ALOX12/urine creatinine levels than the other groups. But there was no difference between the other three groups (Figure 4(B)). The differences were statistically significant. Patient information is shown in Table 1.

**Table 1.** Demographics of patients with hematuria.

Parameter	NC (*n* = 6)	MCD (*n* = 6)	DKD		*p* Value
			DKD1 (*n* = 6)	DKD2 (*n* = 6)	
Age (years)	48.667 ± 12.307	59.000 ± 10.445	55.333 ± 12.176	54.833 ± 9.109	.4251
Sex (F/M)	2/4	2/4	2/4	1/5	.901
U-ACR (mg/g)	11.128 ± 9.740	779.170 ± 493.270*	124.110 ± 91.336	3615.100 ± 2278.700*^,&^	<.01
Serum ALOX12 (ng/mL)	73.403 ± 8.113	72.877 ± 11.387	92.048 ± 4.799**^,#^	111.620 ± 20.224*^,#^	<.01
Urine ALOX12/urine creatinine (ng/mmol)	338.7 ± 81.44	260.2 ± 77.12	340.2 ± 132.3	604.5 ± 174.5**^,###,&&^	<.001

NC group: the normal patients; MCD group: patients with microscopic renal disease patients; DKD1 group: the diabetic kidney disease patients with UACR <300 mg/g; DKD2 group: the diabetic kidney disease patients with UACR ≥300 mg/g; U-ACR: urine-albumin/urine creatinine ratio.

vs. NC *p < .05, **p < .01; vs. MCD ^#^*p* < .01, ^###^*p* < .001; vs. DKD1 ^&^*p* < .05; vs. DKD1 ^&&^*p* < .01.

To visualize the diagnostic efficacy of ALOX12 on the onset and progression of renal injury in DKD from a pathological perspective, six samples from the NC group, 10 from the MCD group, 17 from the DKD1 group, and 25 from the DKD2 group were collected. The demographic information of the subjects is shown in Table 2.

**Table 2.** Baseline patient demographics and renal tissue ALOX12 protein expression.

Parameter	NC (*n* = 6)	MCD (*n* = 10)	DKD		*p* Value
			DKD1 (*n* = 17)	DKD2 (*n* = 25)	
Age (years)	51.500 ± 11.540	47.900 ± 18.180	52.820 ± 7.748	52.600 ± 10.000	.932
Sex (F/M)	2/4	5/5	1/16	6/19	.097
ALOX12 (%)	8.436 ± 0.785	7.433 ± 1.970	13.130 ± 3.458***^,###^	17.400 ± 4.908***^,###,&^	<.001

vs. NC ***p < .001; vs. MCD ^###^*p* < .001; vs. DKD1 ^&^*p* < .05; the protein expression level of ALOX12 was expressed as AOD (%).

**Figure 5.**
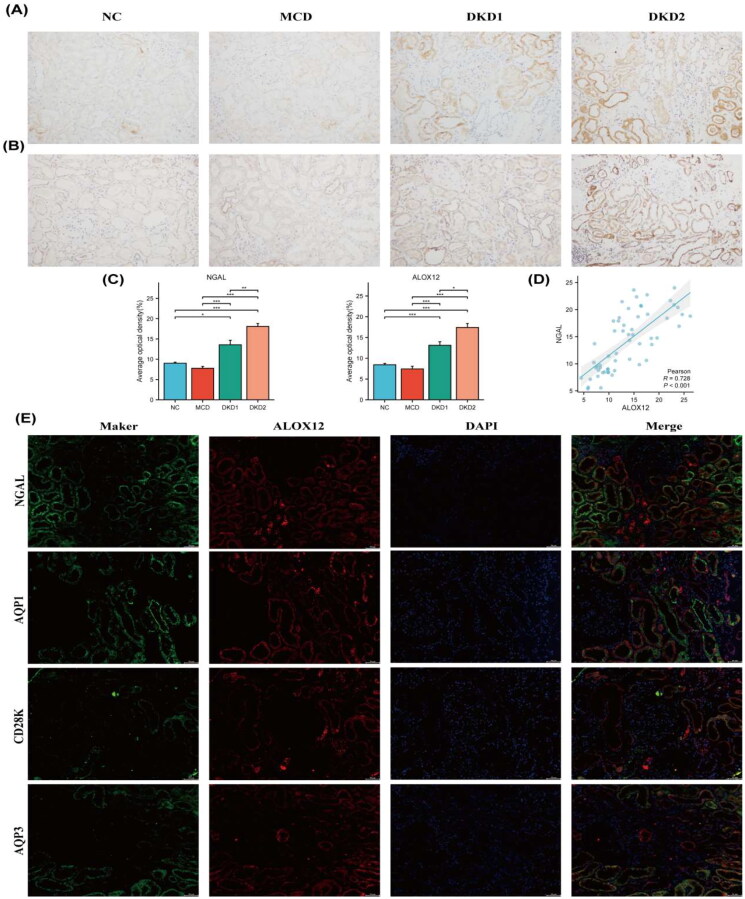
Expression of ALOX12 protein in human kidney tissue. (A) IHC detection of NGAL protein expression in human kidney tissue. (B) IHC detection of ALOX12 protein expression in human kidney tissue. (C) Expression results of ALOX12 levels and NGAL levels. (D) Correlation analysis of ALOX12 levels with NGAL levels. (E) IF double staining of ALOX12 and specific tubular markers in renal biopsy tissues of patients with DKD. Scale bar = 50 μm. *p < .05, **p < .01, and ***p < .001.

Renal tissue IHC staining (Figure 5(B)) revealed that the expression of ALOX12 protein was mainly found in the renal tubules and was increased in DKD patients with macroalbuminuria (*p* < .05). Semiquantitative results showed that ALOX12 levels were higher in the DKD1 group than in the control group (*p* < .05). The level of ALOX12 expression was positively correlated with the expression of NGAL, which is a marker of renal tubular injury [24], with a correlation coefficient of *r* = 0.728 (Figure 5(A,D)). The expression results of ALOX12 and NGAL are shown in Figure 5(C). These findings confirm that the ALOX12 expression level is substantially elevated in DKD and has the potential to be a marker of tubular injury progression in DKD. ALOX12 and NGAL were significantly expressed in the same area, as revealed by double IF staining, indicating that ALOX12 may be implicated in the beginning and development of renal tubular injury. Then, we selected kidney tissues for double IF staining for cellular markers of ALOX12 and renal tubule-resident cells to confirm the exact location of its expression. ALOX12 was clearly expressed in both proximal tubules (marked as aquaporin 1), the collective tubes (marked as aquaporin 3) [25], and the distal tubules (marked as calbindin-D28k) [26] (Figure 5(E)). This suggests that changes in ALOX12 occurred in the renal tubules during the progression of DKD, suggesting that the AA metabolic process occurred in the renal tubules.

### Clinical biochemical and pathological data of patients with DKD

The clinical data are shown in Table 3. The analysis showed that albumin (ALB), total cholesterol (TC), high-density lipoprotein cholesterol (HDLC), low-density lipoprotein cholesterol (LDLC), apolipoprotein A (Apo A), and apolipoprotein B (Apo B) levels were increased in DKD patients with macroalbuminuria (*p* < .05), indicating a higher lipid metabolic burden in DKD. In the DKD2 group, the levels of several urine proteins (including urinary retinol-binding protein (U-RBP), urinary cystatin c (U-CYC), urinary creatinine (U-Cr), and U-ACR) were markedly elevated.

**Table 3.** Clinical data of patients with DKD.

Parameter	DKD		*t*(*Z*)	*p* Value
	DKD1 (*n* = 17)	DKD2 (*n* = 25)		
DM duration (years)	8.441 ± 5.396	8.940 ± 6.604	−0.258	.798
BMI	26.500 ± 3.532	26.320 ± 3.712	0.151	.881
BUN (mmol/L)	8.420 (6.985, 9.395)	9.206 ± 3.354	(–.602)	.547
CRE (µmol/L)	119.200 ± 27.180	135, 500 (115.1, 191.9)	(–1.627)	.104
UA (µmol/L)	390.200 ± 114.40	365.800 ± 87.720	0.780	.440
eGFR, mL/(min*1.73 m^2^)	60.000 (52.000, 115.000)	49.880 ± 25.620	(–2.576)	.010
HbA1c (%)	6.900 (6.450, 7.315)	7.200 (6.300, 8.400)	(–.744)	.457
ALB (g/L)	42.200 (39.350, 43.000)	34.46 ± 7.866	(–2.460)	.014
TC (mmol/L)	4.006 ± 1.121	5.465 ± 1.612	−3.232	.002
TG (mmol/L)	2.110 (1.215, 3.430)	1.590 (1.200, 2.300)	(–1.012)	.311
HDLC (mmol/L)	0.943 ± 0.258	1.174 ± 0.339	−2.373	.023
LHDL (mmol/L)	2.038 ± 0.785	3.312 ± 1.634	−3.370	.002
VLDL (mmol/L)	0.780 (0.400, 1.105)	0.710 (0.450, 1.370)	(–.372)	.710
Apo A (g/L)	1.115 ± 0.178	1.290 ± 0.290	−2.224	.032
Apo B (g/L)	0.794 ± 0.182	1.110 (0.925, 1.305)	(–3.872)	.000
U-RBP (mg/L)	0.460 (0.270, 1.200)	5.500 (2.360, 8.220)	(–3.524)	.001
U-CYC (mg/mL)	0.150 (0.009, 0.200)	0.670 (0.265, 2.040)	(–3.345)	.001
Ucr (mmol/L)	11.59 ± 5.901	7.028 ± 2.726	2.979	.007
U-ACR (mg/g)	73.200 (24.660, 120.800)	2762.000 ± 1955.000	(–5.445)	.000

BUN: blood urea nitrogen; CRE: serum creatinine; UA: uric acid; eGFR: estimated glomerular filtration rate; HbA1c: hemoglobin A1c; ALB: albumin; TC: total cholesterol; TG: triglyceride; HDLC: high-density lipoprotein cholesterol; LDLC: low-density lipoprotein cholesterol; VLDL: very low density lipoprotein; Apo A: apolipoprotein A; Apo B: apolipoprotein B; U-RBP: urinary retinol-binding protein; U-CYC: urinary cystatin c; U-Cr: urinary creatinine; U-ACR: urine-albumin/urine creatinine ratio.

Next, the pathological data were compared. The results demonstrated that in patients with macroalbuminuria, there was thickening of the glomerular basement membrane, proliferation of MC, severe interstitial fibrosis and tubular damage, and a significant increase in both interstitial inflammation and arteriolar hyalinosis (Table 4).

**Table 4.** Pathological data of patients with DKD.

Parameter	DKD		*Z*	*p* Value
	DKD1 (*n* = 17)	DKD2 (*n* = 25)		
Glomerular lesions, *n* (*n*, %)			−4.179	.000
I	0 (0.000)	0 (0.000)		
II				
IIa	13 (76.471)	4 (16.000)		
IIb	3 (17.647)	4 (16.000)		
III	1 (5.882)	13 (52.000)		
IV	0 (0.000)	4 (16.000)		
IFTA, *n* (*n*, %)			−3.156	.002
Score 0	1 (5.882)	0 (0.000)		
Score 1	9 (52.941)	4 (16.000)		
Score 2	7 (41.765)	16 (64.000)		
Score 3	0 (0.000)	5 (20.000)		
Interstitial inflammation, *n* (*n*, %)			−2.215	.027
Score 0	1 (5.882)	0 (0.000)		
Score 1	16 (94.118)	20 (80.000)		
Score 2	0 (0.000)	5 (20.000)		
Arteriolar hyalinosis, *n* (*n*, %)				
Score 0	1 (5.882)	0 (0.000)	−2.063	.039
Score 1	9 (52.941)	7 (28.000)		
Score 2	7 (41.765)	18 (72.000)		
Arteriosclerosis, *n* (*n*, %)				
Score 0	0 (0.000)	0 (0.000)	−.695	.487
Score 1	12 (70.588)	15 (60.000)		
Score 2	5 (29.412)	10 (40.000)		

IFTA: interstitial fibrosis and tubular atrophy.

### Correlation analysis of renal tubular ALOX12 protein levels and clinicopathological features

To assess the clinical relevance of the ALOX12 protein in the renal tubules, we correlated the semiquantitative IHC results of ALOX12 with the clinical biochemical and pathological features of the patients (Table 5). Renal tubular ALOX12 protein levels were positively correlated with lipid metabolism indicators, including TC, HDLC, LHDL, and Apo B. ALOX12 was also positively correlated with indicators of kidney damage, including U-RBP, U-CYC, and U-ACR, where U-RBP is an indicator of renal tubular damage [27].

**Table 5.** Correlation analysis of renal tubular ALOX12 protein levels and clinical and pathological data.

Parameter	*r*	*p* Value
DM duration (years)	−.098	.538
BMI	−.006	.970
BUN (mmol/L)	−.169	.286
CRE (µmol/L)	−.037	.816
UA (µmol/L)	−.084	.595
eGFR, mL/(min*1.73 m^2^)	.046	.774
HbA1c (%)	0.074	.639
ALB (g/L)	−.109	.491
TC (mmol/L)	0.438**	.004
TG (mmol/L)	0.186	.238
HDLC (mmol/L)	.327*	.034
LHDL (mmol/L)	.316*	.041
VLDL (mmol/L)	.263	.092
Apo A(g/L)	.177	.262
Apo B (g/L)	.405*	.008
U-RBP (mg/L)	.412**	.007
U-CYC (mg/mL)	.374*	.015
Ucr (mmol/L)	−.088	.577
UACR (mg/g)	.432**	.004
Glomerular lesions	.211	.181
IFTA	.347*	.024
Interstitial inflammation	.317*	.041
Arteriolar hyalinosis	.088	.578
Arteriosclerosis	−.039	.807

BUN: blood urea nitrogen; CRE: serum creatinine; UA: uric acid; eGFR: estimated glomerular filtration rate; HbA1c: hemoglobin A1c; ALB: albumin; TC: total cholesterol; TG: triglyceride; HDLC: high-density lipoprotein cholesterol; LDLC: low-density lipoprotein cholesterol; VLDL: very low density lipoprotein; Apo A: apolipoprotein A; Apo B: apolipoprotein B; U-RBP: urinary retinol-binding protein; U-CYC: urinary cystatin c; U-Cr: urinary creatinine; U-ACR: urine-albumin/urine creatinine ratio; IFTA: interstitial fibrosis and tubular atrophy.

* *p* < .05.

** *p* < .01.

The findings also revealed that changes in ALOX12 protein expression levels were positively correlated with IFTA scores and interstitial inflammation but not with glomerular lesions, arteriolar hyalinosis, and arteriosclerosis. Further evidence supports the use of ALOX12 as a marker of renal tubular damage.

### Analysis of the diagnostic value of renal tubular ALOX12 for DKD progression

#### Binary logistic regression analysis

In order to determine potential risk factors, univariate logistic regression analysis was initially applied to every clinical biochemical and pathological data point. We employed stepwise regression to control risk variables due to a sample size restriction. The three variables chosen for the multivariate regression analysis were glomerular lesions (*β* = 1863, *p* = .005), ALOX12 (*β* = 0.395, *p* = .046), and U-Cr (*β* = –0.285, *p* = .052). Glomerular lesions and ALOX12 were the model that predicted macroalbuminuria in DKD most accurately; this model excluded U-Cr (*p* > .05). The prediction model was as follows: In(*p*/1 – *p*) = –8.157 + 1.930 × glomerular lesions + 0.318 × ALOX12 (Table 6). In this formula, *p* stands for the chance that the group will belong to group 1 (DKD2 group), and 1 – *p* stands for the likelihood that the group will belong to group 0 (DKD1 group).

**Table 6.** Binary logistic regression analysis.

Variables	Univariate analysis		Multivariate analysis	
	*p*	OR (95% CI)	*p*	OR (95% CI)
ALOX12	.010	1.059–1.535	.046	1.007–2.186
eGFR	.040	0.941–0.999	–	–
ALB	.024	0.799–0.984	–	–
TC	.008	1.244–4.208	–	–
HDLC	.037	1.193–268.120	–	–
LDLC	.012	1.187–3.891	–	–
Apo A	.048	1.028–947.893	–	–
Apo B	.002	10.822–35518.698	–	–
U-RBP	.009	1.093–1.869	–	–
U-CYC	.033	1.210–93.704	–	–
Cr	.009	0.628–0.937	.052	0.564–1.002
Glomerular lesions	.000	2.257–17.542	.005	1.751–23.723
Arteriolar hyalinosis	.038	1.076–12.789	–	–
IFTA	.004	1.831–24.649	–	–

eGFR: estimated glomerular filtration rate; ALB: albumin; TC: total cholesterol; TG: triglyceride; HDLC: high-density lipoprotein cholesterol; LDLC: low-density lipoprotein cholesterol; Apo A: apolipoprotein A; Apo B: apolipoprotein B; U-RBP: urinary retinol-binding protein; U-CYC: urinary cystatin c; U-Cr: urinary creatinine; IFTA: interstitial fibrosis and tubular atrophy.

#### ROC curve analysis

**Figure 6.**
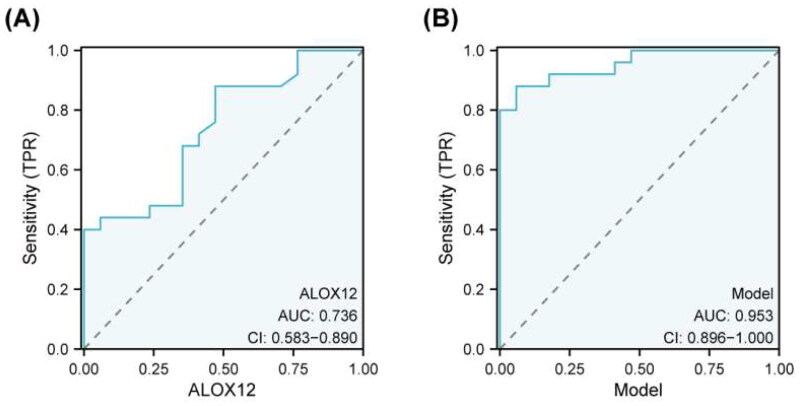
ROC curve analysis to determining diagnostic value. Area under the ROC curve (95% confidence interval (CI)) for the presence of the AUC for macroalbuminuria.

We investigated the diagnostic value of renal tubular ALOX12 protein in patients with macroalbuminuria using ROC analysis. To create ROC diagnostic models, we used the DKD2 group as positive samples. The AUC value of ALOX12 was 0.736 (95% CI 0.583–0.890, *p* = .010). Moreover, the predictive model also showed a good predictive value (AUC = 0.953, *p* < .001) (Table 7). The ROC curves are displayed in Figure 6.

**Table 7.** ROC curve analysis.

	Cut off	AUG (95% CI)	*p* Value	Sensitivity	Specificity	Youden Index
ALOX12	12.342	0.736 (0.583–0.890)	.010	0.880	0.529	0.409
Model	0.613	0.953 (0.896–1.000)	.000	0.880	0.941	0.821

Model: In(*p*/1 – *p*) = –8.157 + 1.930 × glomerular lesions + 0.318 × ALOX12.

## Discussion

This genomics-based study identified the association between ALOX12 and the renal tubular lesions in DKD, and the diagnostic value of ALOX12 was further investigated. The results of the initial transcriptome sequencing of db/db and db/m mouse were used as the basis for genomic analysis. Data analysis suggests that the AA metabolic pathways of lipid metabolism may be closely associated with the development of renal injury in DKD. AA metabolism is inhibited when kidney injury occurs, leading to significant changes in renal blood flow and the vascular filtration rate [28]. For further and more in-depth study, we performed a cluster analysis of DEGs of the AA metabolic pathway and conducted PPI network analysis (Figure 2(C)). The ALOX12 gene and ALOX12e gene were found to be hub genes, making them the leading candidates. We chose ALOX12 for our follow-up research because ALOX12e is an isoform of ALOX12 [23].

Of all genes in the AA pathway, ALOX12 is present in multiple cells of renal tissue and is present in MCs, podocytes, glomeruli, and tubules [29]. 12-LOX is produced during the hyperglycemic response and has a significant impact on development. In earlier investigations, ALOX12 in DKD found that the activation of the 12-LOX and p38 MAPK pathways in podocytes is involved in the development of DKD by mediating extracellular matrix (ECM) synthesis and conversion [30], and that 12-LOX mediates the upregulation of p16ink4a in MCs and glomeruli triggered by HG conditions [31]. 12-LOX participates in the creation of DKD by inducing p21 and p27 protein expression involved in diabetic renal hypertrophy and interacts with angiotensin II to induce p27 upregulation in diabetic kidney tissue [32,33]. However, we explained the clinical importance of ALOX12 from a pathological perspective because the diagnostic importance of ALOX12 in the clinic remains uncertain. Other datasets used for validation have revealed the high diagnostic value of ALOX12 for DKD.

Our experimental results show that ALOX12 levels are significantly increased in the blood of DKD patients but not during disease progression. Besides, urine ALOX12 was increased only in macroalbuminuria patients. Therefore, in order to study the diagnostic efficacy of ALOX12, we can only start from the pathological point of view, which can also visualize the diagnostic efficacy of ALOX12 in DKD kidney injury. In the present study, ALOX12 was primarily found in the renal tubules according to IHC staining (Figure 5(B)). ALOX12 expression in the DKD2 group was significantly different from that in the DKD1, MCD, and NC groups. This finding partially supports that ALOX12 may be associated with DKD onset and dynamic changes, and it may become a promising target for future treatment approaches for DKD. 12-LOX can metabolize AA into 12 HpETE and 12-HETE [34], and the available research indicated that in DM2 patients, exogenous 12 HpETE increases platelet p38-MAPK. This, as a result, encourages platelet activation in pathophysiological states associated with oxidative stress [35] and exacerbates renal injury [36]. Some studies have demonstrated that 12-LOX and 12(S)-HETE have proinflammatory effects [37], Furthermore, 12-LOX metabolites not only limit the maturation of immature dendritic cell DCs but also lessen TLR2- and CD40-mediated cytokine production [38]. This pro-platelet activation, as well as the pro-inflammatory effects of 12-LOX, may contribute to the development of DKD. Thus, it was hypothesized that 12-LOX might also promote the onset of diabetes and, thus, its progression to the complication of DKD.

Renal tubular cells have been demonstrated to be essential in many pathophysiological processes, such as hypertension, and in the fibrogenic process that accompanies CKD [39]. Our research revealed an association between ALOX12 with NGAL, U-RBP, and IFTA. In patients with DKD, renal tubular injury has a bad prognosis and IFTA is a known risk factor for this [40], while NGAL and U-RBP are indicators of renal tubular damage [27]. In previous studies, in monocytes, high levels of HETE activated PPARγ [41], and the latter increased CD36 expression. Increased CD36 expression induced tubular interstitial cell apoptosis and ultimately tubular inflammation and fibrosis [42]. These results suggest that ALOX12 may be a marker of the progression of tubular injury in DKD. Moreover, IF double staining revealed that ALOX12 was expressed at high levels in the proximal tubules and the distal tubules of the kidney and expressed at low levels in the collecting ducts (Figure 5(E)). These results suggest that AA metabolism occurs in most parts of the renal tubules.

The current research focused on assessing the significance of renal tubular ALOX12 expression in the progression of urinary protein disorders. We found significant differences in lipid metabolism in biochemical indicators between the two DKD groups. In previous studies, HETE was shown to activate PPARα [43], which plays a part in renal inflammation through its involvement in regulating lipid metabolism [44]. The LOX metabolic pathway in AA metabolism activates the dysregulation of hepatic glucolipid metabolism, leading to the appearance of IR and NAFLD phenotypes. Additionally, abnormal LOX metabolism maybe connected to the growth of many metabolic diseases, including fatty liver, IR, and hyperlipidemia [45]. In our study, ALOX12 protein expression was positively correlated with TC, HDLC, LDLC, and Apo B. Additionally, these results offer a fresh perspective on how lipid metabolism affects the development of tubular injury in DKD.

As mentioned above, significantly different indicators can be used as biomarkers of DKD progression. These findings imply that ALOX12 may be an influential factor in the progression of DKD. To clarify the diagnostic efficacy of ALOX12 for macroalbuminuria in DKD, we performed an ROC curve analysis to calculate the diagnostic efficacy of ALOX12 (Figure 6(A)). ALOX12 and glomerular lesions were identified as independent risk factors for DKD by binary logistic regression analysis. TA good combined diagnostic value for macroalbuminuria was shown by the regression model's AUC of 0.953 (Figure 6(B)). This offers a novel concept for the future control and treatment of DKD.

However, the small sample size of this study still needs to be improved. In conclusion, through transcriptome sequencing data, we found that ALOX12 expression levels differ in the mouse model of DKD, and this was validated in human kidney tissue. Furthermore, renal ALOX12 protein expression levels were correlated with clinical and renal pathology metrics at various stages of DKD. These findings urge further investigation into the molecular mechanisms underlying the development and progression of DKD, which will help identify possible treatment targets for the disease.

## Conclusions

A number of clinical chemical and pathological markers were discovered to be associated with the expression of the ALOX12 protein, which was also found to be significantly elevated in the renal tissues of DKD patients. The results of this study can be used to further research DKD therapy and prevention options.

## Supplementary Material

Supplemental Material

## Data Availability

The datasets used and/or analyzed during the present study are available from the corresponding author on reasonable request.
