# Supplementary material for: Elevated ALOX12 in renal tissue predicts progression in diabetic kidney disease
Source: Ren Fail. 2024 Feb 12;46(1):2313182. doi: 10.1080/0886022X.2024.2313182 (PMC10863531; doi:10.1080/0886022X.2024.2313182)
Supplement: Supplemental Material [file IRNF_A_2313182_SM1760.pdf]

**Supplementary table I** **Primer sequences of RT-PCR.**

| **Primer** | **Forward primer** | **Reverse primer** |
|---|---|---|
| ALOX12 | GTGGCTGAGGTCATCGCTGTTG | GTAACGGATGTGTGGAACGAGGAG |
| ß-actin | GGACTTCGAGCAAGAGATGG | AGCACTGTGTTGGCGTACAG |

**Supplementary table Ⅱ  Renal function data in mice**

| | **db/m** | **db/db** | ***P*** |
|---|---|---|---|
| **Blood glucose(mmol/L)** | 7.550±0.914 | 25.570±4.234 | <0.001 |
| **24h Urine albumin（mg/24h）** | 7.322±2.550 | 178.500±25.430 | <0.001 |
| **Serum Creatinine(μmol/L)** | 17.580±2.625 | 61.110±9.557 | <0.001 |
| **Serum BUN(mmol/L)** | 15.290±1.532 | 27.380±2.644 | <0.001 |
